# Rural community pharmacist willingness to dispense Suboxone® - A secret shopper investigation in South-Central Appalachia

**DOI:** 10.1016/j.rcsop.2021.100082

**Published:** 2021-10-23

**Authors:** Grace Trull, Erin Major, Chase Harless, William Zule, Bayla Ostrach, Delesha Carpenter

**Affiliations:** a University of North Carolina at Chapel Hill, Eshelman School of Pharmacy, 301 Pharmacy Lane, CB#7355, Chapel Hill, NC 27599, USA; b UNC Health Sciences at Mountain Area Health Education Center, 121 Henderson Road, Asheville, NC 28803, USA; c Research Triangle Institute International, 3040 East Cornwallis Road, Research Triangle Park, NC 27709, USA; d Boston University, School of Medicine, 72 East Concord St, Boston, MA 02118, USA; e University of North Carolina at Chapel Hill, Eshelman School of Pharmacy, 1 University Heights, CPO 2125, Asheville, NC 28804, USA

**Keywords:** Buprenorphine, Opioid use disorder, Pharmacist, Access, Rural

## Abstract

**Background:**

Buprenorphine access is limited for patients with opioid use disorder, especially in rural areas. Telephone audits have identified pharmacist limitations to the dispensing of buprenorphine particularly in independent pharmacies in comparison to chain pharmacies and in rural areas. The objective of this study was to assess rural community pharmacists' stated willingness to dispense buprenorphine-naloxone, and document potential bias and or stigma that the shopper experiences when asking about buprenorphine- naloxone.

**Methods:**

To assess pharmacist willingness, a telephone audit of 15 rural Appalachian North Carolina pharmacies was conducted. Three secret shopper scenarios were utilized including one shopper posing as a new patient, one shopper posing as an out of state patient, and one shopper first asking about buying syringes. Encounters were noted by willingness to dispense buprenorphine, and shoppers were to note any potential stigma and or bias that they experienced while asking for Suboxone®.

**Results:**

Overall, 60% of pharmacies audited indicated willingness to dispense buprenorphine without reservation, and 31% indicated willingness to dispense only under certain circumstances. Pharmacies tended to add more conditions to dispensing to the out of state patient (46%), such as only dispensing if the practitioner was from in state in comparison to the other shopper scenarios. Potential stigma and bias were encountered in 40% of the 45 encounters.

**Conclusion:**

Although pharmacies overall seemed willing to dispense, nuances regarding who pharmacies are most likely to dispense are felt in rural areas. Buprenorphine access limitations were more common in independent pharmacies and more often placed on patients from out of state. Pharmacy- directed education is necessary to reduce stigma and bias and increase patient access to buprenorphine.

## 1 Background

According to provisional data from the Centers for Disease Control and Prevention (CDC), over 81,000 drug overdose deaths occurred in the United States between May 2019 and May 2020; the highest number of overdose deaths ever recorded in a 12-month period.^1^ The CDC cites an influx of synthetic opioids (primarily fentanyl) and the disruption of life due to the COVID-19 pandemic as the basis for this acceleration in overdose deaths.^1^ Geographic disparities in overdose deaths exist, with rates of drug overdose deaths rising in rural areas, and even surpassing urban areas.^2^ In 2017, Appalachian counties had an overdose rate that was 72% higher than in non-Appalachian counties throughout the country.^3^ Medications for opioid use disorder (MOUD), specifically buprenorphine products are an evidence-based treatment shown to reduce opioid overdoses.^4^

Access to buprenorphine products is limited, especially in rural areas, with over half of all rural counties (51%) lacking a provider who is waivered to prescribe buprenorphine.^5^ The Comprehensive Addiction and Recovery Act expanded the range of healthcare providers in the United States (U.S.) that can undergo the training required to apply for the DEA waiver to prescribe buprenorphine, was intended to increase access to buprenorphine providers in rural areas. Although there may be more providers, pharmacy staff can knowingly or unwittingly present barriers to buprenorphine access, with some pharmacists limiting buprenorphine access based on perceived “DEA caps”, concern for misuse/ diversion, buprenorphine knowledge gaps, stigmatizing attitudes toward opioid use disorder (OUD) treatment, and mistrust between pharmacists and physicians.^6^, ^7^, ^8^, ^9^, ^10^, ^11^ A secret shopper study of 704 pharmacies in urban Texas found that only 42.2% of pharmacies were willing to dispense a 1-week supply of generic suboxone, a buprenorphine product.^12^ Among the pharmacies studied, 45% of chain pharmacies versus 12% of independent pharmacies were willing to dispense a 1- week supply of Suboxone®. Research conducted on 13,375 first-time buprenorphine patients indicated that 29.6% had at least one change in pharmacy, and that this change resulted in increased incidence of seven-day gaps in therapy.^13^ A gap of seven days or longer in buprenorphine treatment places patients at risk of opioid- related harm.^13^^,^^15^ In an even more recent secret shopper phone audit of pharmacies in counties with a high opioid overdose rate, one in five pharmacies were unable or unwilling to dispense buprenorphine, with barriers to buprenorphine access more common at independent pharmacies and pharmacies in southern states.^16^

Previous literature indicates that individuals with substance use disorder face stigma in the clinical setting.^16^^,^^17^ According to focus groups conducted of individuals in recovery from opioid use disorder in rural Maine, pharmacists and pharmacy technicians were the most frequent perpetrators of stigma.^18^ Patients in Appalachia have also noted how stigma negatively affected access to behavioral health services including harm reduction programs.^19^ Without access to harm reduction programs, people who inject drugs may seek syringes at their local pharmacy. According to North Carolina law, pharmacists are allowed to sell syringes to anyone without a prescription; however, some pharmacists may refuse to sell syringes, citing informal or formal store policies, such as requiring a prescription on file for an injectable drug.^14^ This deterrent strategy may lead to an increase in communicable disease such as human immunodeficiency virus (HIV) and Hepatitis C.^14^ A recent statement from the American Pharmacists Association encourages revisions to laws and regulations to the unrestricted sale or distribution of sterile syringes and needles by or with the knowledge of a pharmacist in an effort to decrease the transmission of bloodborne diseases.^21^

The objective of this study was to use a secret shopper methodology to simulate patient experiences with accessing buprenorphine in rural community pharmacies in the Appalachian south and to assess how stigma and bias may impact buprenorphine dispensing. This research adds to previous literature by illustrating potential patient-level barriers to buprenorphine access in the rural south and documenting potential stigma and bias exhibited by pharmacist.

## 2 Methods

The cross-sectional qualitative “secret shopper” study described here was conducted from January to March 2021 as a subset of a larger year-long study investigating factors in buprenorphine dispensing at community pharmacies in rural Western North Carolina, a South-Central Appalachian region.^10^^,^^22^^,^^23^ For the purpose of the larger study, “rural” is defined according to a combination of the *Index of Relative Rurality* and U.S. Census rural designations to most accurately capture the combination of topography, infrastructure, travel time to available services, and population distribution.^24^

### 2.1 Human subjects protection

Because study investigators were at multiple institutions, the study underwent dual review by the Institutional Review Boards at both University of North Carolina-Chapel Hill, which determined it exempt from further review, and the Mission Hospital Institutional Review Board, which determined it not human subject's research.

### 2.2 Sampling strategy

Using purposive sampling based on earlier phases of research and regional contextual information, 15 pharmacies out of the 24 pharmacies in a rural region of South-Central Appalachian North Carolina were selected as sites for virtual secret shopper visits (phone calls). Pharmacies were included if they were: located in one of two rural western North Carolina counties where the local health departments prescribe buprenorphine; and/or had a pharmacist participant in an interview about buprenorphine dispensing for an earlier study aim; and/or had been identified by health department staff or prescribers as refusing to fill a buprenorphine prescription for a patient.

### 2.3 Secret shopper preparation & data collection

Data were collected by a team of three trained secret shoppers, in this case, phone auditors, who called the same 15 pharmacies. Each secret shopper presented a unique standardized scenario, resulting in a total of 45 observations. Prior to beginning data collection, the first three authors practiced and refined enacting their scenarios with each other and a community pharmacist. Practice continued until the presentation seemed authentic. Additionally, secret shoppers practiced using the observation guides during recorded practice scenarios.

The standardized scenarios were developed based on existing literature about pharmacist stigma,^6^^,^^7^^,^^11^ and findings from an earlier phase of the study about what local rural pharmacists indicated were the most common reasons they did not dispense buprenorphine.^23^ Each scenario targeted a common reason that could increase pharmacists' reluctance to dispense buprenorphine: the person was a new patient who had not been seen at the pharmacy before (Scenario 1), the patient was from out-of-state (Scenario 2), and the patient was also seeking syringes (Scenario 3). During all encounters the shoppers asked to speak with a pharmacist.

In the first scenario, the secret shopper represented themself simply as a person with a new prescription for buprenorphine (Suboxone®), inquiring if it could be filled at that pharmacy if the script was sent to the pharmacy. The first shopper stated, “I *was wondering, if a doctor sent you a prescription for Suboxone®, could you fill it?*”

In the second scenario the secret shopper represented themself as an out-of-state patient, but from an area directly bordering one of the counties where the pharmacies being called were located, and within about an hour's drive or less from the other county. This is a common scenario for the region where the calls were made, as many patients come from neighboring states to receive OUD treatment. Several pharmacists interviewed in the earlier phase of research specifically mentioned hesitation to dispense to patients from that particular area, even if the prescriber were in-state. The second shopper, who had an accent from the local area, stated *“I live in [specific nearby town across the border], and I was wondering, if a doctor sent you a prescription for Suboxone®, could you fill it?*”

The third scenario, based on existing literature about pharmacist stigma toward pharmacy syringe sales and a few comments about active drug users in the earlier pharmacist interviews, was scripted to probe for potential stigma that OUD patients may face if they signal that they may inject substances in addition to being prescribed buprenorphine.^22^The third shopper opened by asking “*Do you sell syringes?*” and after hearing the answer, followed up by asking “*I have another question, if a doctor sent you a prescription for Suboxone®, could you fill it?*”

For all scenarios, if the pharmacist asked the secret shopper how they would pay or if they had insurance, all three secret shoppers stated they would pay cash. If the pharmacist asked the secret shopper who their prescriber was, secret shoppers stated they were in the process of establishing care (but at a clinic in the community where the pharmacy is located).

### 2.4 Measures

Secret shoppers used a standardized observation guide to record key information about each call. The guides were developed by the second, third, and fourth authors; who are all experienced qualitative researchers.

#### 2.4.1 Willingness to dispense

After completion of each call, secret shoppers immediately completed the observation guide to document the pharmacist's stated willingness to dispense buprenorphine. Willingness was measured on a 3-point scale: −1 (*not willing*), 0 (*willing under certain circumstances*) or 1 (*willing*). Secret shoppers also took notes about any conditional factors that could impact willingness. Conditional factors were any limitations to the dispensing of buprenorphine, for example if pharmacists would only dispense to patients with local providers or to insured patients.

#### 2.4.2 Stigma or bias

Secret shoppers also scored specific indicators of stigma or bias they perceived or observed during their phone interaction on a 3-point scale: 0 (no bias/stigma detected),1 (possible bias/stigma detected), 2 (bias/stigma detected). Stigma and bias were assessed for the following indicators: only dispense under certain circumstances; questioning the legitimacy of the prescription and/or prescriber; won't dispense to cash-paying or uninsured patients; hesitation or tone of voice change when asked for Suboxone®; hesitation or tone of voice change after shopper mentioned they were a new patient; hesitation or tone of voice change after the shopper mentioned they were an out-of-state patient; and answer changes when the shopper mentioned syringes. These metrics were chosen based on existing literature and findings from pharmacist interviews in the earlier study phase.^23^

### 2.5 Data analysis

Each secret shopper took observational, qualitative notes during and immediately after calls; these were thematically coded and analyzed primarily to assign ranking in the indicator categories. All three secret shoppers met after completing five calls to compare coding approaches and reconcile any discrepancies in use of the observation guides. Coders negotiated until reaching 100% agreement, with the second author, an experienced qualitative researcher, guiding the negotiation and overall analysis process. After all observation guides were coded and indicator scoring results tallied, the first and second authors met iteratively to finalize analysis and interpret the data. This analysis included categorizing dispensing willingness by scenario and pharmacy type; and determining average bias/stigma scores by pharmacy and by scenario. Findings were ultimately validated and further interpreted and triangulated by the fourth and senior authors.

## 3 Results

A total of 45 observations were made. In our purposive sample, 6 local independent pharmacies^1^ and 9 commercial chain pharmacies were contacted. Eight of the pharmacies were locations from which a pharmacist had been interviewed in the earlier study phase. Two pharmacies were contracted to dispense buprenorphine to patients from the local health department. Three of the pharmacies were locations where OUD patients and/or buprenorphine prescribers had previously reported encountering difficulty with or being refused a buprenorphine prescription fill.

### 3.1 Willingness to dispense

For the new patient scenario, 12 pharmacy respondents (80%) expressed willingness to dispense buprenorphine; three (20%) expressed willingness under certain circumstances; and none expressed being wholly unwilling to dispense (Table 1.1). Regarding the three circumstances, one pharmacy had a cap on the total number of patients on the tablet formulation of buprenorphine and naloxone but stated that they may be able to dispense sublingual films. One pharmacy noted that they would dispense buprenorphine as long as the prescription was “valid” and after careful examination of the prescription on hand. One pharmacy was embedded in a local clinic, and limited buprenorphine prescriptions filled to only those written by their provider, thus limiting their willingness to dispense.

**Table 1.1:** Pharmacy respondent stated willingness to dispense by patient scenario and type of pharmacy.

	Type of pharmacy	Willing	Willing under certain circumstances	Unwilling
*Scenario 1: New Patient*	*Independent (n* *=* *6)*	6 (100%)	0	0
	*Chain (n* *=* *9)*	6 (67%)	3(33%)	0
*Scenario 2: Out-of-State Patient*	*Independent (n* *=* *6)*	1 (17%)	3 (50%)	2 (33%)
	*Chain (n* *=* *9)*	4 (44%)	4 (44%)	1 (11%)
*Scenario 3: Patient Asking About Syringes*	*Independent (n* *=* *6)*	4 (67%)	2 (33%)	0
	*Chain (n* *=* *9)*	6 (67%)	2 (22%)	1 (11%)
Total	(*n* = 45)	27 (60%)	14 (31%)	4 (9%)

For the out-of-state patient scenario, 5 (33%) pharmacy respondents expressed willingness to dispense buprenorphine without hesitation; seven (47%) expressed willingness to dispense under certain conditions, and three (20%) expressed unwillingness to dispense. Of the seven pharmacy respondents that stated they would only dispense under certain circumstances, four stated the prescription had to be from an in-state provider and three said they would need to verify the prescription.

For the syringe-seeking scenario,10 (67%) pharmacy respondents expressed willingness to dispense without hesitation; four (27%) expressed willingness to dispense under certain conditions, and one (7%) expressed unwillingness to dispense. Of the three respondents who stated they would only dispense buprenorphine under certain conditions, one that stated buprenorphine was out of stock. Of note, this same pharmacist had been willing to order more for a ‘new patient’. Two others stated they would be able to dispense as long as the buprenorphine was prescribed by an in-state provider.

### 3.2 Observed stigma/bias

Bias and stigma were most frequently observed in the syringe-seeking scenario. More than half the time (60%), this secret shopper detected indicators of possible bias and stigma; as compared to the out-of-state patient scenario secret shopper who detected indicators of possible bias and stigma less than half the time (40%); and the new patient scenario secret shopper (20%) (Table 2.1). It is worth noting that none of the secret shoppers observed indicators of explicit stigma and bias that they could directly attribute to their questions about buprenorphine product dispensing or the characteristics of their hypothetical scenario, but each observed some indicators of possible bias or stigma.

**Table 2.1:** Observed stigma/bias in secret shopper pharmacy encounters, by secret shopper scenario.

Secret shopper scenario	Pharmacy type	Explicit stigma/bias observed	Possible stigma/bias observed	No bias/ stigma observed
*Scenario 1: New Patient (n* *=* *15)*	Independent	0/6	1/6 (17%)	5/6 (83%)
	Chain	0/9	2/9 (22%)	7/9 (78%)
	Total	0/15	3/15 (20%)	12/15 (80%)
*Scenario 2: Out of State Patient (n* *=* *15)*	Independent	0/6	3/6 (50%)	3/6 (50%)
	Chain	0/9	3/9 (33%)	6/9 (67%)
	Total	0/15	6/15 (40%)	9/15 (60%)
*Scenario 3: Patient Asking About Syringes (n* *=* *15)*	Independent	0/6	4/6 (67%)	2/6 (33%)
	Chain	0/9	5/9 (56%)	4/9 (44%)
	Total	0/15	9/15 (60%)	6/15 (40%)

As seen in Table 2.1, of independent pharmacies called, potential stigma and bias was observed in one call (17%) regarding a new patient, half the time for an out-of-state patient, and in 67% of the calls regarding a patient seeking syringes. In comparison, among chain pharmacies, potential stigma was observed in two calls (22%) regarding new patients, one-third of calls regarding out-of-state patients, and two-thirds of calls regarding a patient asking for syringes.

In the new patient scenario, the secret shopper observed indicators of stigma and/or bias in three encounters. Two pharmacies questioned the legitimacy of the prescription or of the prescriber, one questioning whether the provider was qualified to send in Suboxone®, and asking who the prescriber was. One pharmacy was noted to have a clear tone change when asked about “Suboxone®,” and indicated they would fill the prescription “as long as it was valid.”

In the out-of-state patient scenario, the secret shopper observed indicators of stigma and/or bias in 6 encounters. These included hesitation/ tone of voice change upon hearing the caller was an out-of-state “patient,” (*n* = 6). In two scenarios the pharmacy staff placed the caller on hold to ask another pharmacist for their perspective/ whether or not they would be able to dispense. In one encounter, the pharmacist let out a long sigh and paused before noting that they would be able to dispense as long as the prescription was from an in-state doctor.

Sixteen observations of possible stigma and/or bias were documented for the syringe scenario, including nine pharmacy respondents that hesitated or changed their tone of voice when asked if they would fill a Suboxone® prescription after being asked about selling syringes. Five of fifteen respondents stated they would only dispense Suboxone® under certain circumstances after being asked about syringe sales, including one stating “*As long as it's not from out of state*,” and another remarking” If your primary care is here and your doctor here prescribes it, then yes, we will fill it.” Two of 15 pharmacies reported they require the prescription to be from certain providers; another required the prescription to come from the local health department with which they have a dispensing contract. Another pharmacy did not specify the prescriber(s), just that they need to be local. One of 15 pharmacies indicated they were “*locked in*” (sic) and unable to take on any additional buprenorphine patients.

### 3.3 Syringe stigma

Eight of fifteen pharmacies expressed unwillingness to dispense syringes without the patient having a prescription on file for an injectable medication such as insulin or testosterone, despite the legality of syringe sales without a prescription in North Carolina. Some of the responses from pharmacists when asked for syringes included, “*Like…. For insulin?*” and “*Uh yes, we do, but you just have to have a prescription on file with us to verify you're taking insulin or testosterone or something like that.*” Many of these responses included a notable hesitancy and/or a change in tone, with stigma and bias were experienced in 5 of these 8 encounters with pharmacies that noted they only sold to patients that had a prescription on file for syringes. One pharmacist said with a pointed tone: “*We sell* insulin *syringes.”* Two pharmacists responded tersely but affirmatively: “*Ummmm yeah, we do;*” “*We do, for our diabetic patients.”*

## 4 Discussion

Findings from this study indicate an overall willingness to dispense Suboxone®, but this willingness is nuanced and differs depending on certain characteristics of both the patient and the pharmacy. These findings are consistent with an earlier phase of this study that noted pharmacists' preference to dispense buprenorphine prescriptions from known prescribers and to know patients.^23^ Staff at four of the fifteen pharmacies expressed willingness only to dispense to an out-of-state patient if the prescription was from an in-state provider. For comparison, in an earlier phase of the larger study, pharmacists from a quarter of the same locations mentioned hesitation to dispense to out-of-state patients.^23^ This research aligns with a survey of 102 pharmacists, 73.5% of pharmacists noted they were not at all likely to fill a prescription for buprenorphine written by an out-of-state practitioner.^6^ It is important to note that there are currently no state or federal regulations that require the practitioner to be from in-state. In rural Appalachia, the nearest pharmacy to a patient may be located across state lines, and with these informal policies limiting dispensing, patients may not have access to buprenorphine. Without access to buprenorphine at their local pharmacy, patients are at increased risk of opioid-related harm.^14^^,^^15^ Further research should explore why pharmacists implement these strategies and how to best provide patients with the care they need.

Independent pharmacies in this study were more likely than the commercial chain pharmacies to dispense buprenorphine only under certain conditions. This aligns with previous secret shopper studies that noted buprenorphine access barriers were more pronounced among independent pharmacies when compared to chain pharmacies.^25^ The caller from out of state was more likely to experience stigma and bias in an independent pharmacy (50%) in comparison to chain pharmacies (33%). Additionally, this research indicates that a shopper who first asks about syringes experienced stigma and or bias in both independent and chain pharmacies. Education tailored to pharmacists in practice regarding how to best communicate with patients with substance use disorder is necessary to reduce stigma and increase pharmacist confidence in serving this population.^27^

Previous literature indicated that pharmacies' failure to stock buprenorphine can limit access for patients.^12^ Although it is difficult to ascertain the rationale behind why pharmacies fail to stock buprenorphine, it has been posited that it is due to perceived DEA imposed ordering limits. Research indicates that the ordering limits that are imposed on pharmacists are not set directly by the DEA, but instead by medication distributors.^10^ This limitation by pharmacy distributors was described by the pharmacist to secret shoppers in this study by explaining they were “locked-in” to a certain number of buprenorphine patients and could not take on more patients. This is concerning because delays in buprenorphine treatment can place the patient at risk of opioid- related harm.^13^ In order to reduce this barrier for patients, pharmacy stakeholders and distributors should work together to ensure that regulatory guidance is clearly communicated to pharmacy staff.

This study indicated that greater than half of rural Appalachian pharmacies surveyed were not willing to dispense syringes without a prescription for injectable drugs on file. Previous literature on Indiana pharmacies indicated a similar trend in that half of pharmacies surveyed did not sell syringes without a prescription, and pharmacies in areas of high opioid overdose mortality were 56% less likely to sell syringes without a prescription than those in communities with lower rates.^26^ In encounters in which pharmacies refused to sell syringes to patients without a prescription, indications of stigma and bias were common, which may be limiting the sale of syringes in the rural south. Education of pharmacists including the importance of selling syringes without a prescription is necessary to reduce communicable diseases such as human immunodeficiency virus and hepatitis.

### 4.1 Stigma

The prevalence of perceived stigma in the form of such metrics as hesitation or a change in the tone of voice indicates that many of the participants hold biases against patients prescribed buprenorphine. The scenario that recorded the most instances of possible stigma and bias was the patient that first asked for syringes before asking about the availability of Suboxone®, which indicates that this bias may be more prevalent against patients who are perceived as using substances recreationally. Shoppers documented perceived stigma and bias more commonly in independent pharmacies in comparison to chain pharmacies. This research indicates that pharmacist- directed education regarding communication and harm reduction strategies is necessary to reduce stigma and bias surrounding opioid use disorder.

The relationship between stigma and willingness to dispense is complex and additional factors, such as store level policies, may have influenced dispensing. For example, there were several instances in which the pharmacist was willing to dispense buprenorphine even though stigma was detected. This may be because the store had a policy that supported buprenorphine dispensing. More research to explore additional factors that could influence willingness to dispense is warranted.

### 4.2 Limitations

Although the telephone design was necessary given the COVID-19 pandemic and the necessity to avoid unlawful representation of a prescription, it is possible that the willingness of pharmacists to dispense buprenorphine may be different if the patient had been physically in the pharmacy. Due to the hypothetical nature of the telephone encounter when no buprenorphine could truly be dispensed, no indicators of explicit bias were observed. A second limitation noted is that the three secret shoppers may have coded pharmacists' communication differently. In an attempt to limit coding differences, the coders met twice during the coding process to compare how they would code certain conversations. Additionally, coders wrote notes about their interactions to discuss how to best code interactions. To mitigate recall bias, the researchers documented their results on the observation guides immediately following each encounter. While coders worked to limit coding differences, they were unable to ensure that each coder would speak to the same pharmacy member. In each encounter however, the coders asked to speak with the pharmacist. One pharmacy had a contract limiting which prescribers they can accept and dispense medications from, likely impacting data on their willingness to dispense. A secret shopper study necessarily produces data based on what pharmacists say they will do, not what actually takes place when medication dispensing is sought in real life; thus, these findings may not reflect the real-world experiences of patients filling buprenorphine prescriptions as demonstrated in a forthcoming manuscript about patient experiences at these same pharmacies. However, the results likely simulate what real patients would experience if they called pharmacies to inquire about filling a buprenorphine prescription. Although we sampled 62.5% of pharmacies in our area, purposive sampling of pharmacies with a history of refusing to fill buprenorphine may have led us to overestimate stigma. Given that the 15 pharmacies studied represent 62.5% of the pharmacies in the two-county region, this research is valuable for addressing the local pharmacy micro-climate in rural Appalachia.

### 4.3 Directions for future research

Identification of the rationale behind the preference for in-state prescribers may be useful to guide development of practical tools and education to optimize and expand buprenorphine access at rural community pharmacies. In particular, training that addresses the benefits of increasing buprenorphine access and the harms of delaying buprenorphine fills is warranted. Training about buprenorphine caps and state laws around syringe sales also could impact pharmacist willingness to dispense buprenorphine. Additionally, future research should explore the current status of syringe sales at pharmacies and patients' ability to access syringes with or without a prescription. This study could be conducted in other rural communities to determine if these results can be replicated in other areas of the country.

## 5 Conclusion

Consistent with other secret shopper studies documenting barriers to buprenorphine dispensing in Southern and rural community pharmacies, pharmacists in this study of two South-Central Appalachian counties were least likely to express willingness to dispense buprenorphine for patients from out-of-state in comparison to new patients, and somewhat more hesitant to express willingness to dispense to patients that first asked about syringe sales. Conditions placed on the dispensing of buprenorphine included that patient and/or prescribers needed to be in-state, and pharmacy agreements to only fill for certain prescribers. Ordering limitations (real and perceived) also affected willingness to dispense. Indicators of potential stigma and bias were most commonly encountered by a secret shopper first asking about syringe sales. Findings from this research suggest a need for tailored education for pharmacists around communicating with patients with opioid use disorder, and harm reductions strategies to prevent communicable diseases. Such training should focus on addressing barriers to dispensing for out-of-state patients as well as ways to reduce stigma and bias, including for syringe sales and buprenorphine dispensing for patients perceived to be people who inject drugs.

Although efforts to reduce bias among pharmacists are critical, it can be a slow process. Meantime, harm reduction organizations could begin collecting information from clients or from pharmacies regarding policies and practices surrounding sales of syringes and buprenorphine and distributing the information to clients during in-person interactions and via their websites. Prescribers with buprenorphine waivers could be notified regarding potential issues filling their prescriptions and provided information about how to overcome potential barriers to filling buprenorphine prescriptions that they could pass on to their patients. These measures could help reduce barriers to syringes and buprenorphine while stigma reduction efforts are underway.

## Funding

This study was jointly funded by 10.13039/100013056UNC Eshelman School of Pharmacy and 10.13039/100005563RTI.

## Declaration of Competing Interest

There are no competing interests to declare.

## Footnotes

1 Including one embedded in a local clinic.
